# General practitioners’ perspectives on management of early-stage chronic kidney disease: a focus group study

**DOI:** 10.1186/s12875-018-0736-3

**Published:** 2018-06-06

**Authors:** Carola van Dipten, Saskia van Berkel, Wim J. C. de Grauw, Nynke D. Scherpbier-de Haan, Bouke Brongers, Karel van Spaendonck, Jack F. M. Wetzels, Willem J. J. Assendelft, Marianne K. Dees

**Affiliations:** 1 Department of Primary and Community Care, Radboud University Medical Center, PO Box 9101, 6500 HB Postal Route 117, Nijmegen, The Netherlands; 2 Department of Nephrology, Radboud University Medical Center, PO Box 9101, 6500 HB Postal Route 464, Nijmegen, The Netherlands

**Keywords:** Chronic kidney disease, Guidelines, Primary care, Qualitative research, Quality of care

## Abstract

**Background:**

Guideline adherence in chronic kidney disease management is low, despite guideline implementation initiatives. Knowing general practitioners’ (GPs’) perspectives of management of early-stage chronic kidney disease (CKD) and the applicability of the national interdisciplinary guideline could support strategies to improve quality of care.

**Method:**

Qualitative focus group study with 27 GPs in the Netherlands. Three analysts open-coded and comparatively analysed the data. Mind-mapping sessions were performed after data-saturation.

**Results:**

Five themes emerged: defining CKD, knowledge and awareness, patient-physician interaction, organisation of CKD care and value of the guideline. A key finding was the abstractness of the CKD concept. The GPs expressed various perspectives about defining CKD and interpreting estimated glomerular filtration rates. Views about clinical relevance influenced the decision-making, although factual knowledge seems lacking. Striving to inform well enough without creating anxiety and to explain suitably for the intellectual ability of the patient caused tension in the patient-physician interaction. Integration with cardiovascular disease-management programmes was mentioned as a way of implementing CKD care in the future. The guideline was perceived as a rough guide rather than a leading document.

**Conclusion:**

CKD is perceived as an abstract rather than a clinical concept. Abstractness plays a role in all formulated themes. Management of CKD patients in primary care is complex and is influenced by physician-bound considerations related to individual knowledge and perception of the importance of CKD. Strategies are needed to improve GPs’ understanding of the concept of CKD by education, a holistic approach to guidelines, and integration of CKD care into cardiovascular programmes.

**Trial registration:**

Not applicable**.**

**Electronic supplementary material:**

The online version of this article (10.1186/s12875-018-0736-3) contains supplementary material, which is available to authorized users.

## Background

Chronic kidney disease (CKD) is an important healthcare problem. The estimated prevalence in the Netherlands is 12% [1], which is similar to the prevalence in the US and the UK (13%) [2, 3]. CKD causes substantial morbidity and mortality, mainly related to increased cardiovascular risk [4–6]. It is expected that the number of CKD patients will increase due to aging of the population and increased prevalence of diabetes and hypertension [5, 7].

Most early-stage CKD patients receive care from general practitioners (GPs). Several international guidelines have been developed to improve the quality of care in primary care [8, 9]. In the Netherlands GPs function as gatekeepers and are supported by nurse practitioners in the area of chronic diseases. The Dutch interdisciplinary guideline for CKD (DIG-CKD) [10] for family practice and nephrology provides recommendations for GPs about the identification and management of CKD and serves as a guide for shared care with nephrologists [10]. This guideline was introduced in 2009. It is very similar to the NICE guideline for CKD, is distributed by the Dutch College of General Practitioners and freely available, in print and online.

Despite guideline recommendations, a number of quantitative studies indicate substantial deficiencies in the quality of care delivered, including CKD recognition and monitoring as well as reaching blood pressure targets [11–13]. Several barriers for implementation of CKD management have already been described; objections to the label CKD because of fear, stigmatisation and physiological decrease of kidney function; lack of time; low expectations of management; and guideline familiarity [14–16]. A commonly mentioned solution is education, but GPs who participated in a recent Dutch CKD trial in which they received extra education on CKD management, had difficulties implementing the guideline recommendations [17]. In order to improve the implementation of CKD care, it is important to know GPs’ underlying thoughts and beliefs about CKD and the implementation of the guideline. The present study therefore aims to explore the perspectives of GPs who were familiar with the guideline on CKD management in daily practice. We also examined the applicability of the national interdisciplinary guideline.

## Methods

### Study design

Given the explorative character of the research aim, we considered a qualitative approach to be the most appropriate. We selected a focus group design because perspectives are more likely to be revealed by interaction and discussion with peers. Grounded theory was used as a theoretical framework [18]. We used the consolidated criteria for reporting qualitative health research (COREQ) as a reporting structure [19]. Besides adjustment of the topic list, we made no further modifications of the methods during the study.

### Selection of participants

Four focus group interviews were conducted with 27 Dutch GPs. We recruited (by phone and e-mail) from practices that had participated in the CONTACT study (Consultation Of Nephrology by Telenephrology Allows optimal Chronic kidney disease Treatment in primary care) [20]. These GPs were definitely informed about the guideline. We assumed they used the guide in daily practice, thus being able to provide knowledge about the perspectives of implementation of CKD management. In the Netherlands, GPs are responsible for providing and implementing CKD care. Although Dutch nurse practitioners (NPs) are involved in care programmes for diabetes and cardiovascular disease, their involvement in CKD care is minor. We therefore recruited only GPs. Two GPs were not involved in the CONTACT trial study. They participated because two participants in focus group 4 cancelled at the last minute. They were involved in scientific research and familiar with the guideline.

A purposive stepwise sampling strategy [21] was applied to ensure heterogeneity for gender, age, urbanization, and experience in general practice (Table 1). Sampling, data collection and analysis occurred iteratively. Practice and personal data were collected prior to the interviews. All GPs consented to participation, and they were assured that anonymity and confidentially was guaranteed. No patient data were used.

**Table 1:** Participant characteristics

Sex *n (%)*	
Male	13 (48.1%)
Female	14 (51.9%)
Age in years	
Mean	50
Range	30–62
Working experience in Years	
Mean	19
Range	1–33
Familiarity with DIG-CKD *n* (%)^a^	
Scarce	3 (11.1%)
Reasonable	13 (48.1%)
Good	8 (29.6%)
Very good	3 (11.1%)
Frequency of DIG-CKD usage *n (%)*^b^	
Weekly	3 (11.1%)
Monthly	11(40.7%)
< than once a month	8 (29.6%)
Rarely	4 (14.8%)
Never	1 (3.7%)
Usage of telenephrology	21 (77.8%)
Practice urbanization *n (%)*	
Rural	11 (40.7%)
Urban	16 (59.3%)
Active as practice holder *n (%)*	26 (96.3%)
Presence of a nurse practitioner *n (%)*	27 (100%)

DIG-CKD: Dutch Interdisciplinary Guideline for Chronic Kidney Disease

^a^Subjective perception of the participating GPs

^b^Defined as the minimal use of the guideline

### Data collection

Before starting, a topic list (Additional file 1) was created by reviewing relevant literature and in consultation with the research team and two CKD-patients of the Dutch Kidney Foundation. A senior psychologist (KvS) with extensive experience in medical healthcare and chairing focus group studies moderated the focus groups. Either one of the two investigators (CD or SB), both GP trainees, PhD students and trained in qualitative research, and a research intern (BB) observed the focus groups and noted non-verbal communication and details about group interaction. The sessions lasted 120 min each. After each focus group, the investigator and Chair discussed observations made during the sessions and adjusted the topic list for each following focus group, in discussion with the research team. All focus group discussions were audio-taped and transcribed verbatim. In the analysis of the fourth focus group, no new codes or concepts were found. We decided that saturation had been reached at that moment.

### Data analysis

The transcripts were analysed with the constant comparative analysis method [21] and with the aid of a computer program (Atlas.ti version: 7.1.5). Analyses started after the first focus group interview. The analysts (SB and BB) independently used open and inductive coding. They discussed and merged codes after each focus group. In the case of disagreement, members of the peer group (MD and WG) were consulted. A consensus code list arose, which was used to code the second transcript. New codes were added and discussed as described after each next coded transcript. After three focus group sessions, another researcher (CD) became involved as SB left the research project. This researcher coded all transcripts as a third coder. The research intern (BB) and the second researcher (CD) independently coded the fourth session. After saturation was reached, the codes were sorted into categories and themes. It took five consensus meetings in which members of the research group (CD, SB, BB, MD, NS, and WG) participated to construct the final thematic map. For a detailed description of the analysis process, see Additional file 2. A native-English speaker translated the illustrative quotes.

## Results

### Participants

Four focus group interviews were conducted between November 2014 and March 2016. A total of 147 GPs were invited to participate, of whom 71 responded. Forty-one GPs were interested in participating, while 30 GPs declined, mostly due to lack of time. Altogether, 27 GPs were included by purposive sampling, and 5 to 8 GPs participated in each session. Table 1 presents the characteristics of the participants.

### Overview

Five main themes emerged: 1) defining CKD, 2) knowledge and awareness, 3) patient-physician interaction, 4) organisation of CKD care, and 5) value of the guideline. For a detailed description of codes, categories and themes, see Additional file 3.

### Defining CKD

CKD was experienced as a difficult and abstract concept. CKD seems intangible. The diagnosis is not a clinical one, but is merely based upon laboratory findings without patient complaints and- in the view of participants- in some cases without clinical consequences. The participants struggled to interpret the eGFR values due to eGFR fluctuations and strict cut-off points. Age and physiology were considered relevant to interpret eGFR values, but also whether to label patients with the CKD diagnosis. Participants felt that there was no fixed definition of CKD. Furthermore, whether CKD is a disease on its own or a risk factor for cardiovascular disease, like hypertension, was discussed.

*“The initial question was what is your picture of chronic kidney damage, and honestly, that picture is just a check mark in a row of risk factors.”* (FG1, man, 60-70y)

### Knowledge and awareness

#### Professional competence

Educational gaps in the contents of the guideline and about proteinuria were reported. Nevertheless, there was a shared feeling that awareness of CKD has improved due to increased monitoring of diabetes and cardiovascular disease and the introduction of the DIG-CKD. The recurrent use of the guideline appeared to facilitate a learning curve so that managing CKD patients became easier. This reduced the urge to consult or refer to a nephrologist.

*“Yes, at a certain point you know what the nephrologist will say. If I have heard it a few times, then I think: ok, that’s the next step that I can take with this patient.”* (FG2, woman, 50-60y)

#### Perception of the importance of CKD

Due to insufficient knowledge about the clinical consequences of CKD, treatment and adherence to the guideline were trivialised. There was scepticism concerning health profit for patients if GPs would fully adhered to the guideline. GPs’ decision making was influenced by expectations about poor prognosis and quality of life.

*“I think it’s a difficult problem ... a lot of medication, that influences kidney function. But then I think I’d rather have poor kidney function than be a patient who is extremely short of breath.”* (FG4, woman, 50-60y)

### Patient-physician interaction

#### Informing patients

It appeared difficult to find the best approach for informing patients. The major concern was to inform enough without creating anxiety. Both straightforward communication and metaphors were used to explain the CKD diagnosis.

*“…That there is a kind of rinsing machine in your body that keeps your blood clean, I say then. And if that machine doesn’t work well, then your blood gets poisoned.”* (FG1, man, 60-70y)

Striving to adequately inform patients without creating unnecessary anxiety and to ensure the explanations and education was tailored to the patients educational level was found difficult. The right moment to inform patients and a lack of information material were also discussed.

#### Patient empowerment

The GPs felt the urge to empower patients in managing their CKD, but struggled to provide methods to increase patients health literacy. GPs felt that patients should especially take preventive measures, but they also had doubts about the efficacy of self-management. They felt that gaining patients’ compliance would require time-consuming explanations. Especially in the case of co-morbidity, the balance between energy spent on self-management and the return it would generate worked out negatively.

*“Yes, but that is in the whole of chronic care, it is certainly very difficult because people with kidney function disorders, even not considering age, so often have other problems with smoking, blood pressure, weight, etc.”* (FG4, man, 40-50y)

### Organisation of CKD care

#### Primary care

There was not always consensus regarding GPs’ policies within the practices, though the participants agreed about the importance of congruence of CKD care. The presence of alignments about task delegation to the nurse practitioner varied. There was discussion about the future implementation of CKD care.

*“They usually come into the picture through the annual blood test in chronic-disease management programmes, so that you have already checked them in connection with other disorders.”* (FG1, man, 40-50y)

#### Primary-secondary care interface

The accessibility of nephrologists and the transfer of medical information needs improvement. The participants found it instructive to consult a nephrologist. The preferred method of contacting nephrologists (teleconsultation or by phone) differed.

*“Formerly, specialist were easy to reach, now you get lost in the logistics of the hospital.”* (FG3, man 50-60y)

#### Medical specialists

The views towards nephrologists varied and were mainly based on previous experiences in contact and communication with them. Some had doubts about the added value of nephrologists’ involvement. Losing control over patients’ treatment after referral to a specialist was difficult for the GPs. They considered that other aspects should be taken into account, influencing how aggressively patients should be treated. GPs also experienced one-way communication and held the opinion that nephrologists do not involve GPs enough.

*“I always find it sad when people land at the nephrologists’ and have a blood pressure that is 2 mmHg too high. Then they have to come back three times. While I think: yeah right, boys, yeah. ”* (FG3, man, 50-60y)

### Value of the guideline

#### Facilitators

Accepting the recommendations of the existing interdisciplinary guideline induced a sense of safety. The guideline was used by the GPs to reduce knowledge gaps, resulting in a learning curve. The shared opinion was that the guideline created more awareness for CKD and improved the quality of care for patients.

#### Barriers

The GPs found treatment and referral criteria in the interdisciplinary CKD guideline too strict and precise, which made following the guideline time consuming. Furthermore, a feeling of medicalization of CKD patients was mentioned.

*“What is the use of medicalising someone of great age with everything?”* (FG4, woman, 40-50y)

#### Advice for improvements

According to the GPs more attention should be paid to the context of CKD patients and to how to interpret laboratory and clinical findings, while at the same moment taking the context of the patient into account. More advice about how to enlarge patient empowerment would be helpful.

## Discussion

### Summary of main findings

Perception of CKD as an abstract concept is a key finding in this study. The perceived abstract CKD concept seems to play a role in all formulated themes. It influences the GPs’ experienced confidence on CKD knowledge. Clinical relevance also seems to be lacking, and there is scepticism concerning treatment benefits for patients. GPs act at their own discretion, taking into account patients’ age, prognosis and quality of life. The interdisciplinary guideline is therefore seen as a rough guide rather than leading. The abstractness of the CKD concept forms an obstacle in conveying the CKD concept to patients.

### Comparison with existing literature

#### Abstractness

Previous findings like educational gaps, guideline familiarity, tensions surrounding ICPC (international classification of primary care) labeling and physiology are in line with our study results [14–16, 22–24]. However, the importance of the perceived abstractness of the CKD concept, which in our study was a key finding, has never been highlighted as a central theme, causing difficulties for GPs in managing CKD. Since our findings are based on a study of trained GPs with special interest in CKD we presume that CKD as a concept will be even more difficult for other GPs who have not been trained in CKD explicitly. We indentified several factors that contribute to the abstractness of the CKD concept. Of these factors, renal aging, a diagnosis based on eGFRs and the tension between disease and risk factors have been earlier discussed in studies of Crinson and Simmonds [14, 22]. In our study, another aspect of the abstractness of CKD appeared to be the struggle to interpret eGFRs (i.e. fluctuating eGFRs, eGFR versus severity of CKD). This is a new and fascinating insight, which raises the question of how the interpretation of an eGFR value differs from the interpretation of a blood glucose level. Both a CKD diagnosis and a diabetes diagnosis are laboratory based, have strict cut-off points, and have no symptoms in an early stage. Despite these similarities, diabetes management is well integrated in daily practice in primary care while CKD management is not. In the knowledge that CKD provides as much risk of cardiovascular disease as diabetes does [25], this is a remarkable difference.

#### Education

We have seen that extra education for the GPs in our previous study did not meet their needs in interpreting and managing CKD [20]. We hypothesise that educational interventions should be even more intense, as Pang found in a study in which GPs perceived an increase in knowledge after interventions during which they were personally mentored by nephrologists [26].

#### Patient empowerment

GPs prefer to make CKD patients partners in care, but they encounter several barriers. Patient empowerment is time-consuming, and GPs have doubts about the efficacy of self-management. If patient empowerment is recommended in guidelines, attention should be paid to these barriers.

### Strengths and limitations

The heterogeneity of the participants supported the generalisability of the findings. Internal validity was established through independent coding in triplicate, the use of Atlas.ti and the mind-mapping sessions with the research team in which additional perspectives and interpretation of analysis and findings were discussed. Analysis by three analysts and similar findings from previous other studies helped to triangulate the findings. The rigor of the data is supported by the iterative approach of the focus group and the interim data analysis. Some limitations should be considered. The focus groups were performed in Dutch, so that representative quotations needed translation. This may have caused loss of nuance, which we tried to limit through translation by a native-English speaker. The moderator was a psychologist, which could be a restriction regarding in-depth interviewing in the medical field. Another possible limitation is that most recruited GPs were previous participants in the CONTACT study. This might be related to a special interest in the research theme, possibly influenced their knowledge of and commitment to the subject. However, GPs who are not familiar with the subject may have even more difficulties with CKD care while they have insufficient knowledge about the guideline to provide answers to the research question. In order to avoid analysis bias as much as possible, the research team members differed in profession, age, experience regarding CKD care, and experience as a GP. Some had been involved in previous research on CKD (including the trial), but others had no specific experience in CKD research when the focus group study was performed. All researchers were GPs (in training). These background factors may have influenced our findings, but we can’t indicate the direction of a possible bias.

### Implications for practice

Our study provides insight into the perspectives of GPs concerning early-stage CKD management and could give input for future quality-improvement interventions. A major direction should be to improve GPs’ understanding of the clinical concept of CKD. This could be done by education, which should also focus on clinical relevance, prognostic value of CKD, proteinuria and the interpretation of eGFRs in relation to age and comorbidity. Instructions on how to give a suitable explanation of the CKD diagnosis to patients might as well be part of GPs’ education.

Our opinion is that embedding CKD care in an integrated care programme of all cardiovascular risk factors, including CKD, hypertension and diabetes, may support GPs and patients to maintain an overview. For those who were not diagnosed with diabetes or cardiovascular disease, a comparable care program provides the best chance of creating awareness and improving the quality of care for CKD patients. The tension between disease-specific guidelines and the holistic care preferred by GPs is - besides the abstract concept of CKD - perhaps the most important implementation barrier. GPs wish to maintain a patient-centred approach in providing high-quality CKD care, deviating from guideline recommendations when necessary.

## Conclusions

This paper shows that care for patients with chronic kidney disease in primary care is a complex interplay of an abstract concept and physician-bound considerations. Difficulty interpreting the concept of CKD and doubts about the clinical relevance of CKD in the light of the patient’s personal situation are the main reasons for deviating from guideline recommendations. Quality improvement strategies should focus on education of GPs in CKD-specific knowledge, especially in judging CKD relevance and GP-patient communication. Guidelines should include more guidance in eGFR interpretation, clinical consequences, and suggestions for tailoring interventions to the personal context of the individual patient. GPs feel there is tension between personalised healthcare and CKD-specific guidelines.

## Additional files

Additional file 1: Topic list. A list of relevant topics concerning CKD management which was constructed before start of the study to ensure all research items were covered and discussed in the focus groups. (DOCX 19 kb)

Additional file 2: Analysis details. A detailed description of the iterative process of data collection and analysis. (DOCX 14 kb)

Additional file 3: Codes, Categories and Themes. A detailed table with codes and their description, from which categories and themes originated. (DOCX 37 kb)

## Abbreviations

CKD: Chronic kidney disease
CONTACT: Consultation of nephrology by telenephrology allows optimal chronic kidney disease Treatment in primary care
COREQ: COnsolidated criteria for REporting Qualitative health research
DIG-CKD: Dutch interdisciplinary guideline for chronic kidney disease
eGFR: Estimated glomerular filtration rate
FG: Focus group
GPs: General practitioners
KDIGO: Kidney disease: improving global outcomes
UK: United Kingdom
US: United States

## References

[1] de Zeeuw D, Hillege HL, de Jong PE. The kidney, a cardiovascular risk marker, and a new target for therapy. Kidney Int Suppl. 2005;(98):S25–9. 10.1111/j.1523-1755.2005.09805.x 16108967

[2] Roderick PRM, Mindell J (2011). Prevalence of chronic kidney disease in England: findings from the 2009 health survey for England. J Epidemiol Commun H.

[3] Coresh J, Selvin E, Stevens LA, Manzi J, Kusek JW, Eggers P (2007). Prevalence of chronic kidney disease in the United States. JAMA.

[4] Go AS, Chertow GM, Fan D, McCulloch CE, Hsu CY (2004). Chronic kidney disease and the risks of death, cardiovascular events, and hospitalization. N Engl J Med.

[5] Fox CS, Matsushita K, Woodward M, Bilo HJG, Chalmers J, Lambers Heerspink HJ (2012). Associations of kidney disease measures with mortality and end-stage renal disease in individuals with and without diabetes: a meta-analysis. Lancet.

[6] Matsushita K, van der Velde M, Astor BC, Woodward M, Levey AS, de Jong PE (2010). Association of estimated glomerular filtration rate and albuminuria with all-cause and cardiovascular mortality in general population cohorts: a collaborative meta-analysis. Lancet.

[7] Mahmoodi BK, Matsushita K, Woodward M, Blankestijn PJ, Cirillo M, Ohkubo T (2012). Associations of kidney disease measures with mortality and end-stage renal disease in individuals with and without hypertension: a meta-analysis. Lancet.

[8] Kidney Disease: Improving Global Outcomes (KDIGO) CKD Work Group (2013). KDIGO 2012 Clinical Practice Guideline for the Evaluation and Management of Chronic Kidney Disease. Kidney Inter Suppl.

[9] NICE Clinical guideline: Chronic kidney disease (2008). Early identification and management of chronic kidney disease in adults in primary and secondary care.

[10] De Grauw WJC, Kaasjager HAH, Bilo HJG, Faber EF, Flikweert S, Gaillard CAJM (2009). Landelijke transmurale afspraak chronische nierschade. Huisarts Wetenschap.

[11] Allen AS, Forman JP, Orav EJ, Bates DW, Denker BM, Sequist TD (2011). Primary care management of chronic kidney disease. J Gen Intern Med.

[12] Lenz O, Mekala DP, Patel DV, Fornoni A, Metz D, Roth D (2005). Barriers to successful care for chronic kidney disease. BMC Nephrol.

[13] Van Gelder VA, Scherpbier-De Haan ND, De Grauw WJ, Vervoort GM, Van Weel C, Biermans MC (2016). Quality of chronic kidney disease management in primary care: a retrospective study. Scand J Prim Health Care.

[14] Crinson I, Gallagher H, Thomas N, de Lusignan S (2010). How ready is general practice to improve quality in chronic kidney disease? A diagnostic analysis. Br J Gen Pract.

[15] Blakeman T, Protheroe J, Chew-Graham C, Rogers A, Kennedy A (2012). Understanding the management of early-stage chronic kidney disease in primary care: a qualitative study. Br J Gen Pract.

[16] Abdel-Kader K, Greer RC, Boulware LE, Unruh ML (2014). Primary care physicians' familiarity, beliefs, and perceived barriers to practice guidelines in non-diabetic CKD: a survey study. BMC Nephrol.

[17] van Dipten C, van Berkel S, van Gelder VA, Wetzels JF, Akkermans RP, de Grauw WJ (2017). Adherence to chronic kidney disease guidelines in primary care patients is associated with comorbidity. Fam Pract.

[18] Walker D, Myrick F (2006). Grounded theory: an exploration of process and procedure. Qual Health Res.

[19] Tong A, Sainsbury P, Craig J (2007). Consolidated criteria for reporting qualitative research (COREQ): a 32-item checklist for interviews and focus groups. Int J Qual Health Care.

[20] van Gelder VA, Scherpbier ND, van Berkel S, Akkermans RP, De Grauw IS, Adang EM (2017). Web-based consultation between general practitioners and nephrologists, a cluster randomized controlled trial. Fam Pract.

[21] Marshall MN (1996). Sampling for qualitative research. Fam Pract.

[22] Simmonds R, Evans J, Feder G, Blakeman T, Lasserson D, Murray E (2016). Understanding tensions and identifying clinician agreement on improvements to early-stage chronic kidney disease monitoring in primary care: a qualitative study. BMJ Open.

[23] Vest BM, York TR, Sand J, Fox CH, Kahn LS (2015). Chronic kidney disease guideline implementation in primary care: a qualitative report from the TRANSLATE CKD study. J Am Board Fam Med.

[24] Lo C, Teede H, Ilic D, Russell G, Murphy K, Usherwood T (2016). Identifying health service barriers in the management of co-morbid diabetes and chronic kidney disease in primary care: a mixed-methods exploration. Fam Pract.

[25] Matsushita K, Coresh J, Sang Y, Chalmers J, Fox C, Guallar E (2015). Estimated glomerular filtration rate and albuminuria for prediction of cardiovascular outcomes: a collaborative meta-analysis of individual participant data. Lancet Diabetes Endocrinol.

[26] Pang J, Grill A, Bhatt M, Woodward GL, Brimble S (2016). Evaluation of a mentorship program to support chronic kidney disease care. Can Fam Physician.

