# Supplementary material for: General practitioners’ perspectives on management of early-stage chronic kidney disease: a focus group study
Source: BMC Fam Pract. 2018 Jun 6;19:81. doi: 10.1186/s12875-018-0736-3 (PMC5991428; doi:10.1186/s12875-018-0736-3)
Supplement: Supplementary file 1 — Topic list. A list of relevant topics concerning CKD management which was constructed before start of the study to ensure all research items were covered and discussed in the focus groups. (DOCX 19 kb) [file 12875_2018_736_MOESM1_ESM.docx]

**Additional file 1: Topic list**

**CKD diagnosis:** exploring the vision of GPs about CKD care in primary care. When is it important to pay attention to CKD and why? Has the DIG-CKD changed GPs’ opinion in this regard?

**ICPC coding:** what consideration exists in relation to ICPC coding?

**CKD knowledge:** exploring CKD knowledge and educational needs. Has the DIG-CKD increased your knowledge? What creates awareness of CKD? Has knowledge influenced GPs’ attitude towards CKD?

**Treatment of CKD:** what points do GPs consider important in treating CKD patients and what is the role of the DIG-CKD? What do GPs themselves contribute to CKD treatment? Is there therapeutic nihilism?

**Usage of the DIG-CKD**: decisions of GPs in consultation and referral issues and the role of the DIG-CKD. Are there expectations, arguments or factors that cause deviation from the guideline recommendations? Why does this affect guideline adherence?

**Informing patients:** exploring visions of GPs about communication with CKD patients. What barriers do GPs encounter in communication about CKD? Do GPs believe their patients are well informed? What does the DIG-CKD contribute to communication?

**Practice organisation:** exploring GPs’ visions about the implementation of CKD care in family practice. Who are the people involved in CKD care in family practice and what is their role? What is the role of the nurse practitioner? Are there clear agreements between health care workers?

**Collaboration**: exploring the vision and experiences of GPs concerning collaboration with nephrologists and primary care colleagues. Do the previously mentioned experiences affect GPs in using the guideline? Has the introduction of the DIG-CKD changed collaboration?

**Finances:** exploring what financial factors play a role in CKD care. Explain whether financial issues are barriers or facilitators for CKD care.
