# Supplementary material for: General practitioners’ perspectives on management of early-stage chronic kidney disease: a focus group study
Source: BMC Fam Pract. 2018 Jun 6;19:81. doi: 10.1186/s12875-018-0736-3 (PMC5991428; doi:10.1186/s12875-018-0736-3)
Supplement: Supplementary file 2 — Analysis details. A detailed description of the iterative process of data collection and analysis. (DOCX 14 kb) [file 12875_2018_736_MOESM2_ESM.docx]

**Additional file 2: Analysis details**

| **Focusgroup 1**  October 2014 | Inductive coding of the transcript by SB and BB, using Atlas.ti | Consensus about the codes between SB and BB | Discussion with SB, BB MD and WG about codes, findings and lacks so far | Adjustment of the topic list based on the findings and observations during the focus group session. |
| --- | --- | --- | --- | --- |
| **Focusgroup 2**  November 2014 | Inductive coding of the transcript by SB and BB, using Atlas.ti | Consensus about the codes between SB and BB | Discussion with SB, MD, NS and WG about codes, findings and lacks so far | Adjustment of the topic list based on the findings and observations during the focus group session. GPs demographic information was analysed before inclusion in the next focus group session. |
| **Focusgroup 3**  February 2015 | Inductive coding of the transcript by SB and BB, using Atlas.ti | Consensus about the codes between SB and BB | First consensus meeting with SB, BB, MD and WG about codes, findings and lacks so far. | Adjustment of the topic list based on the findings and observations during the focus group session.. |
| **CD involved**  September 2015 | Coding of the transcripts of focus group 1,2,3 by CD, using Atlas.ti | Consensus of codes by comparing codes of SB/BB and CD by CD and MD | Discussion with the research team about saturation. Second consensus meeting of the new codes and comparison of codes with the transcripts. | Adjustment of the topic list based on the findings so far. |
| **Focusgroup 4**  March 2016 | Coding of the transcript by CD and BB, using Atlas.ti | Consensus about the codes between CD and BB | Discussion with the research team about the codes, findings and saturation. | Preparation of the final consensus meetings, extraction of codes out of atlas.ti |
| **Consensus meetings**  March 2016  April 2016  May 2016 | Third consensus meeting with CD and MD, comparison of codes with the transcripts | Fourth consensus meeting with CD, MD, NS and WG, building categories and themes. Comparison of theme and categories with the transcripts | Final consensus meeting with CD, MD, NS and WG building categories and themes. Comparison of theme and categories with the transcripts |  |
