# Supplementary material for: General practitioners’ perspectives on management of early-stage chronic kidney disease: a focus group study
Source: BMC Fam Pract. 2018 Jun 6;19:81. doi: 10.1186/s12875-018-0736-3 (PMC5991428; doi:10.1186/s12875-018-0736-3)
Supplement: Supplementary file 3 — Codes, Categories and Themes. A detailed table with codes and their description, from which categories and themes originated. (DOCX 37 kb) [file 12875_2018_736_MOESM3_ESM.docx]

**Additional file 3: Codes, Categories and Themes**

| Theme | | |
| --- | --- | --- |
| Category | | |
| Subcategory | Code | Description of the code |

| Defining CKD | | |
| --- | --- | --- |
|  | Abstract concept | Chronic kidney damage (CKD) is an abstract concept based on statistics. |
|  | Definition of kidney damage | General practitioners find it difficult to define kidney damage. |
|  | Disease or risk factor | General practitioners think differently about whether kidney damage is a risk factor or a disease. |
|  | Age-related variability | General practitioners feel a greater need to deal quickly with young patients. |
|  | Physiological changes | General practitioners take physiological changes into consideration when making decisions. |
|  | Sharp cut-off point | Sharp statistical cut-off points are not practicable for general practitioners. |
|  | Borderline cases | General practitioners have difficulty in making policy decisions regarding patients whose kidney function is on the borderline of normal/CKD. |
|  | Progress of kidney function | The progress of kidney function over time is important with regard to decision making. |
|  | Variable kidney function | Variable kidney function is often not an immediate reason for general practitioners to contact a nephrologist. |
|  | Multimorbidity | CKD is one of the many patient comorbidities that general practitioners have to deal with. |
|  | CKD does not produce symptoms | Chronic impaired kidney function does not produce symptoms. |
| Knowledge and awareness | | |
| Professional competence | | |
|  | General practitioners’ knowledge | General practitioners’ knowledge of CKD varies enormously. |
|  | Doubts about medical value of monitoring | General practitioners are unsure whether monitoring CKD is medically useful. |
|  | Ready knowledge of DIG-CKD content | General practitioners indicate that they do not have ready knowledge of DIG-CKD content. |
|  | Understanding of proteinuria | General practitioners have too little knowledge about proteinuria. |
|  | Diagnosis made from a single measurement | In practice, a diagnosis is made on the basis of a single measurement; this is not consistent with the DIG-CKD. |
|  | Experience facilitates deviation from guidelines | Experienced general practitioners are more likely to deviate from the guidelines. |
|  | Knowledge of CKD changes choice of intervention | New knowledge about CKD means that general practitioners see/deal with CKD differently than in the past. |
|  | General practitioners’ awareness | General practitioners indicate they are alert with respect to CKD patients. |
|  | Awareness through education | General practitioners indicate that general training has made them more alert with respect to CKD. |
|  | Awareness through DIG-CKD | General practitioners indicate that the DIG-CKD has made them more alert with respect to CKD. |
|  | Awareness through CVRM | General practitioners indicate that monitoring kidney function as part of other care programmes has made them more alert. |
|  | Awareness through CONTACT | General practitioners indicate that the CONTACT study has made them more alert with respect to CKD. |
|  | Alertness with respect to medical prescriptions | Alertness with respect to medical prescriptions is achieved through ICPC coding and monitoring. |
|  | Polypharmacy | Polypharmacy in patients makes general practitioners alert with respect to monitoring. |
|  | Interest in CKD | General practitioners indicate that they first have to be interested in CKD before studying it in more depth. |
| Perception of the importance of CKD | | |
| Judging CKD importance | Severity of kidney damage | General practitioners think differently regarding the severity of kidney damage. |
|  | Acknowledging the importance of CKD | General practitioners consider it important to give attention to kidney damage during a consultation. |
|  | Trivialisation of CKD | General practitioners sometimes trivialise CKD, also in their explanations to the patient. |
|  | General practitioners avoid dealing with CKD | General practitioners sometimes avoid giving CKD extra attention. |
|  | Workload | General practitioners feel they have an increased workload, which means they sometimes pay less attention to medication doses, do not deal with CKD in a consultation, and sometimes spend too little time explaining CKD to patients. |
|  | Stopping smoking | General practitioners say there is a notion that even greater results can be gained by stopping smoking. |
|  | Gut feeling | General practitioners often rely on their gut feelings. |
| Expectations (incl. therapeutic) | Limited therapeutic consequences | General practitioners say they consider therapeutic consequences to be very limited. |
|  | Preventing a drop in eGFR | General practitioners try to prevent a drop in eGFR as treatment. |
|  | Prevention | General practitioners focus on prevention in the treatment of CKD. |
|  | Monitoring | General practitioners monitor CKD as treatment. |
|  | Health benefits | General practitioners are unsure about the health benefits of DIG-CKD/treatment. |
|  | Quality of life | General practitioners say they consider quality of life to be an important factor when it comes to policy-making for patients with CKD. |
|  | Patient prognosis | Life expectancy and patient prognosis play a role in treatment policy decisions. |
| Behavioural consequences | Following up advice from consultations | It is generally accepted that advice given during a consultation should be followed up thoroughly. |
|  | Deliberate deviation from DIG-CKD | General practitioners deviate from the guidelines deliberately as they see fit. |
|  | DIG-CKD leeway | General practitioners think there is leeway in the guideline for making their own decisions. |
|  | Patient context facilitates deviation | Specific patient contexts enable general practitioners to deviate from the DIG-CKD. |
| Patient-physician Interaction | | |
| Informing patients | | |
|  | Informing patients < 50 | General practitioners inform patients with an eGFR < 50 |
|  | Relationship of trust | General practitioners indicate that they communicate better, and achieve more, with patients they have built a relationship of trust with. |
| Communication skills | Competency in explaining CKD | General practitioners find it difficult to provide explanations tailored to suit the patient. |
|  | Educational skills | General practitioners are unsure about their own ability to educate/inform patients about CKD. |
|  | GPs differ in the degree to which they check whether the patient has understood. | General practitioners differ in the degree to which they check whether the information they have given is clear to the patient. |
| Communication method | Manner of giving a diagnosis | General practitioners differ in the way they give patients a diagnosis (to-the-point, or a ‘softened’ message). |
|  | General practitioners give straightforward diagnosis | General practitioners give the diagnosis in a straightforward manner, do not use metaphors, do not try to soften the message and use words such as ‘kidney damage’. |
|  | Allowing leeway for the patient’s experience | In their communication with patients, general practitioners try to allow leeway for the patient’s own experience which allows them to respond to emotions. |
|  | Use of metaphors | General practitioners use many metaphors when explaining CKD to patients |
|  | NHG patient letter | Use of patient information; NHG patient letter |
|  | Thuisarts.nl | Use of patient information; thuisarts.nl |
| Barriers | Ethnicity | The general practitioner finds diversity in patients’ cultural backgrounds difficult with respect to treating kidney damage. |
|  | Illiteracy | General practitioners find it difficult to provide illiterate patients with a good explanation. |
|  | Causing concern | General practitioners think they may cause patients concern by informing them about CKD. |
|  | Tailor-made explanation | General practitioners try to tailor information to suit the patient’s knowledge/intellect. |
|  | Patient material is not straightforward | The various sources of information/patient material are not straightforward. |
|  | Motivational interviews | The general practitioner uses motivational interviews to include the patient in the problem. |
|  | Non-prescription medicines | Concerns have been expressed about the fact that medication can be bought over the counter. |
| Patient empowerment | | |
|  | Usefulness of self-management | Opinions differ with respect to the usefulness of talking about self-management. |
|  | Self-management is difficult because of the multifactorial aetiology of CKD | General practitioners find it problematic that CKD has such a wide capacity for self-management. |
|  | Patient as healthcare partner | General practitioners consider it essential that the patient also takes responsibility for his/her CKD. |
|  | Patients’ awareness | General practitioners differ in their experience of patient’s own awareness of the diagnosis of kidney damage. |
|  | Patients’ own capacity important with respect to self-management | The patient’s own capacity is an important factor in whether self-management succeeds or fails. |
|  | Provision of information | Making sure the patient is well-informed is important for successful self-management. |
|  | Expectations of the patient | The patient’s own expectations play a role in self-management and the choice of treatment policy. |
|  | Access to medical records | If patients have access to their own medical records, this motivates general practitioners to ensure that efficient care/explanation is provided. |
|  | Media influences patient awareness | General practitioners have noticed that the media also increases patient awareness. |
| Organisation of CKD care | | |
| Finances | | |
|  | Finance is not a reason to follow the DIG-CKD | General practitioners indicate that financial issues do not influence the way they work or whether they follow the DIG-CKD. |
|  | Funding from health insurers | No good agreements have been made with health insurers regarding CKD patients and financial compensation for general practitioners. |
|  | Patients’ financial capacity | Patients sometime incur expenses, which may affect the treatment policy. |
| Primary care | | |
|  | Logistical feasibility | There are concerns as to whether it is practically feasible to follow the DIG-CKD strictly for all CKD patients in general practice. |
| Alignment between GPs | Agreements within general practice | Agreements between general practitioners and nurse practitioners differ. |
|  | Continuity of care | General practitioners think that continuity of care contributes to good CKD care. |
|  | Knowledge of colleagues’ policies | Knowledge varies among general practitioners with respect to how their colleagues deal with kidney damage in practice. |
|  | Policy varies among GPs | Policy can sometimes vary between the different general practitioners within one practice. |
|  | Coding for handover to colleagues | Coding is important for handing over care to fellow general practitioners in normal and out-of-hours surgeries. |
|  | Coding eGFR <60 | An agreement has been made to use a code (ICPC) for an eGFR < 60. |
|  | Coding eGFR <50 | An agreement has been made to use a code (ICPC) for an eGFR < 50. |
| Alignment with nurse practitioners | Nurse practitioners take over some tasks | Nurse practitioners see many CKD patients as part of existing programmes (CVRM and diabetes) and take over some tasks from general practitioners. |
|  | CKD is medically complex for nurse practitioners | Some general practitioners consider the care for CKD patients too complex for nurse practitioners. |
|  | Varying degree of visibility with respect to activities nurse practitioner | General practitioners say they do not always know whether nurse practitioners include CKD in their policy. |
|  | Nurse practitioners stringent with respect to prevention | Nurse practitioners follow protocols meticulously, and also apply this to the patients. |
| Alignment with pharmacists | Agreements with pharmacy | General agreements between general practitioners and pharmacies differ. |
|  | Reporting eGFR to pharmacy | General practitioners report eGFR values to pharmacy if this is relevant. |
|  | Role of pharmacy with respect to dosage | The pharmacy adjusts medication dosage according to kidney function. |
|  | Mutual agreements between pharmacies | Pharmacies do not always have mutual agreements. |
| ICT-related organisation | Including ICPC code on medical prescriptions | Including the ICPC code on medical prescriptions increases transparency with respect to the choice of a particular medication/dosage. |
|  | ICPC coding essential | ICPC coding is essential. |
|  | ICPC coding for locums | ICPC coding is important so that locums can also provide tailor-made care. |
|  | Medication pop-up | General practitioners consider medication pop-ups useful following coding of kidney damage. |
|  | ICPC coding increases alertness | ICPC coding makes general practitioners more alert to CKD. |
|  | ICPC coding for CKD | An extra ICPC code is required for patients with a mild form of mild CKD. |
|  | HIS | In order to support the care of CKD patients as effectively as possible, many improvements could be made in the general practitioners information system (in Dutch: HIS). |
|  | HIS combined with KIS | KIS and HIS do not go well together and are regarded as an obstacle. |
|  | No up-to-date eGFR at out-of-hours surgery | No up-to-date eGFRs are available at the out-of-hours surgery. |
| Lab | Lab experiences lack of uniformity for eGFR results. | General practitioners have noticed that the results of an eGFR < 60 differ with each measurement per lab and sometimes within the same lab; they regard this as a hindrance. |
|  | KCL lab provides list of CKD patients | Some practices receive a list of all recent kidney function test results from the lab. |
| Implementation of CKD care | Kidney damage given attention as part of other care programmes. | Monitoring of kidney damage is included in existing care programmes. |
|  | Integration with other care programmes | General practitioners have difficulty integrating CKD in care programmes for other comorbidities. |
|  | Regional agreements | GPs say there is little need for a separate regional agreement in addition to the DIG-CKD. |
|  | Integrated care | GPs think differently about the added value of integrated care for kidney damage. |
|  | NHG standard | GPs think differently about the necessity of a NHG standard for CKD. |
| Primary/secondary care interface | | |
|  | GPs lose sight of patient | General practitioners find they lose sight of CKD patients once they have been referred to secondary care. |
|  | Actively inviting patients to return | General practitioners occasionally ask patients to come back for a consultation, or adapt their consultation question. |
|  | Experience with nephrologist does not influence use of DIG-CKD | General practitioners say that negative experiences with the nephrologist are no reason to deviate from the DIG-CKD. |
|  | The DIG-CKD has changed the collaboration with nephrologists to a varying degree | GPs think differently about the influence of the DIG-CKD on their collaboration with nephrologists. |
|  | Transfer to primary care | General practitioners think that healthcare provided as secondary care can often be provided as primary care. |
| Transfer of medical records | Privacy rules | Privacy rules obstruct the exchange of information between relevant health care providers. |
|  | Handover of patient information | Handover of patient information does not always take place smoothly; this hinders the provision of good health care. |
| Logistical matters | Agreement with nephrologists | General practitioners indicate that they sometimes make agreements that fall outside of the guidelines in the interests of the patient. |
|  | Telenephrology facilitates cooperation | General practitioners think that telenephrology has been beneficial with respect to cooperation. |
|  | GPs/specialists have various positions/tasks | General practitioners and specialists have different tasks and positions with respect to the care provided for kidney damage. |
|  | Accessibility of nephrologist | Nephrologists are sometimes difficult to reach for consultations; GPs find this annoying. |
|  | Knowing the nephrologist improves cooperation | Cooperation is easier if the specialist knows the nephrologist. |
| Nephrologists’ consultation | Consultation is useful | General practitioners consider the information they acquire during a consultation is both useful and usable. |
|  | Learning effect of consultation | General practitioners experience a learning effect having had several consultations with a nephrologist. |
|  | Consulting for advice about medication | General practitioners consult nephrologists primarily for advice about medication. |
|  | Consulting by phone | General practitioners indicate a preference for consultations by phone. |
|  | Consulting by telenephrology | General practitioners indicate a preference for consultations by telenephrology. |
| Medical specialists | | |
|  | Capacity of secondary care | General practitioners don’t think secondary care would have adequate coping capacity if all patient referrals were sent to this level. |
| View on nephrologists | Varying experiences with nephrologists | General practitioners have differing experiences of working with nephrologists |
|  | Added value of nephrologists | General practitioners often (but not always) feel that referral to a nephrologist has no added value. |
|  | Nephrologists take a different view | Nephrologists look at kidney damage differently than general practitioners. |
|  | Experience/attitude of nephrologist | The attitude sometime taken by nephrologists plays a role in collaborations with GPs. |
|  | Nephrologists do not take patient context into account | General practitioners think that nephrologists do not take patient context adequately into account. |
|  | One-way collaboration | General practitioners have the impression that they work together with the specialist, but that this is not always reciprocated. |
|  | General practitioners uncertain about nephrologists’ efforts | General practitioners feel uncertain about whether nephrologists do their best to provide good care. |
|  | Nephrologists are medically precise in their work | General practitioners differ in their opinions regarding the precision of nephrologists’ policies. |
| Between specialists | Lack of alignment between specialists | The policies of nephrologists, cardiologists and geriatricians differ and there is no mutual alignment. |
|  | Difficult collaboration with cardiologists | General practitioners find the collaboration with cardiologists extremely difficult. |
| Value of the guideline | | |
| Facilitators | | |
|  | DIG-CKD as intermediate step | The DIG-CKD is regarded as a good intermediate step between a referral and making one’s own policy. |
|  | DIG-CKD and awareness | The DIG-CKD has promoted awareness among general practitioners. |
|  | DIG-CKD as a line of approach | The DIG-CKD is considered to be a good line of approach. |
|  | DIG-CKD increases knowledge | Using the DIG-CKD increases general practitioners’ knowledge. |
|  | DIG-CKD reassures doctor | Following the DIG-CKD provides reassurance for general practitioners. |
|  | DIG-CKD is practical | The DIG-CKD is regarded as practical. |
|  | DIG-CKD is EBM | The DIG-CKD is evidence-based medicine, so why deviate from it? |
|  | DIG-CKD provides opportunity to use expertise | The DIG-CKD provides the opportunity to make use of expertise, also when consultation is advised. |
| Barrieres | | |
|  | Medicalisation | General practitioners feel that following the DIG-CKD to the letter results in the medicalisation of patients. |
|  | DIG-CKD causes unnecessary referrals | General practitioners feel that the DIG-CKD advises referrals too quickly. |
|  | DIG-CKD is a strict protocol | The DIG-CKD is regarded as a strict protocol that allows little flexibility. |
|  | DIG-CKD is time-consuming | Using the DIG-CKD is time-consuming. |
|  | DIG-CKD becoming redundant | The need to refer to the DIG-CKD is continually diminishing due to the learning effect. |
| Advice for improvements | | |
|  | DIG-CKD and frequency of check-ups | The DIG-CKD does not indicate how frequently certain patients must be checked. |
|  | DIG-CKD and patient context | The DIG-CKD does not take patient context into account. |
|  | DIG-CKD and ethnicity | The DIG-CKD does not take into account the differences between ethnic groups. |
|  | Complexity of calcium and PTH in DIG-CKD | General practitioners find the information on calcium, phosphate and PTH complicated in the DIG-CKD. |
|  | DIG-CKD and patient participation | There are no tips/tricks in the DIG-CKD with which to make the patient partial ‘owner’ of the problem. |
|  | DIG-CKD and layout | Flow diagrams in the DIG-CKD are complex, and there is too much text. |
|  | No differential diagnosis in DIG-CKD | General practitioners say they miss a good differential diagnosis and other diagnostic criteria for other kidney conditions. |
|  | DIG-CKD lacks detail on proteinuria | The role of proteinuria is not explained clearly enough in the DIG-CKD. |
|  | Not enough practical tips in DIG-CKD | General practitioners think the DIG-CKD does not provide enough practical tips on things like salt, vitamin D and patient information. |
|  | DIG-CKD not helpful in informing patient | General practitioners feel the DIG-CKD has not helped them in knowing how to inform patients. |
|  | DIG-CKD lacks information on dealing with fluctuating kidney function values | General practitioners indicate that the LCA lacks information about how to deal with strongly fluctuating kidney functions, including kidney functions > eGFR 60. |
|  | DIG-CKD lacks information about expected effects of intervention | The DIG-CKD does not provide information about what effects may be expected, and when, following an intervention. |
|  | The DIG-CKD does not distinguish between acute and chronic renal insufficiency | General practitioners find it difficult to identify acute renal insufficiency. |
|  | GPs do not wish to see role of informal carer included in DIG-CKD | General practitioners do not consider it necessary to include the role of informal carer in CKD guidelines. |

**Abbreviations**

CKD Chronic kidney disease

CONTACT Consultation Of Nephrology by Telenephrology Allows optimal Chronic kidney disease Treatment in primary care

DIG-CKD Dutch interdisciplinary guideline for chronic kidney disease

eGFR Estimated glomerular filtration rate

GPs General practitioners

HIS Electronic medical record

ICPC International Classification of Primary care

KIS Electronic medical record for chronic care programmes
